# Pre-hospital transported patients - a resource for accessing prognostic risk factors

**DOI:** 10.1186/1757-7241-23-S1-A2

**Published:** 2015-07-16

**Authors:** Camilla LN Bech, Mikkel Brabrand, Annmarie Lassen

**Affiliations:** 1 Department of Emergency Medicine, OUH Odense University Hospital, Odense, Denmark; 2 Department of Emergency Medicine, Sydvestjysk Sygehus, Esbjerg, Denmark

## Background

The survival of patients transported by ambulance depends not only on the clinical condition but also on other patient-related factors and the organizational pre-hospital setup. Until now, information on patients in the pre-hospital system has been almost unexplored. However, these data could form a valuable resource for assessing potential risk factors associated with adverse outcomes. Our aim was to describe ambulance transports to the Emergency Department and identify prognostic factors accessible in the pre-hospital phase and associated with seven-day mortality.

## Methods

We included all adult patients (> 18 years) transported by ambulance to the Emergency Department at Odense University Hospital in the period 1^th ^of April 2012 to 30^th ^of September 2013. Ambulance personnel recorded vital signs and other clinical findings on a structured form on paper during the ambulance transport. Each contact was linked to information from population based healthcare registers in order to identify comorbid conditions and information on mortality. Demographic factors and first registered vital sign were analyzed by univariate logistic regression analysis with seven-day mortality as the outcome.

## Results

In total, 24,620 ambulance contacts were identified. The median age of the patients was 61.4 years (IQR: 46-79), 49.5% were female, and 34.4% had severe comorbidity (defined as a Charlson Comorbidity Index > = 2). Over-all, seven-day mortality was 3.9% (95% CI: 3.6-4.1%), with 450 deceased females (3.9%) and 499 deceased males (4.0%). Univariate analyses revealed age, above 70 years; OR 16.2 (95% CI: 8.61-30.3), Charlson score ≥2; OR 2.65 (95% CI 2.28-3.09), and vital parameters outside normal reference to be associated with seven-day mortality: Glasgow Coma Scale score < 14 OR 8.62 (95% CI: 7.52-9.89), peripheral oxygen saturation < 95% OR 2.75 (95% CI 2.41-3.15), respiratory rate >20/min OR 5.15 (95% CI: 4.5-5.91), systolic blood pressure<110mmHg OR 2.17 (95% CI:2.17-3.35) and pulse > 90 OR 2.47 (95% CI: 2.16-2.83), respectively.

## Conclusions

We found that several pre-hospital-registered vital signs recorded by ambulance personnel at first contact with the patient were prognostic factors of seven-day mortality.

